# Localized Combination Therapy Using Collagen–Hydroxyapatite Bone Grafts for Simultaneous Bone Cancer Inhibition and Tissue Regeneration

**DOI:** 10.3390/polym17162239

**Published:** 2025-08-18

**Authors:** Alina Florentina Vladu, Madalina Georgiana Albu Kaya, Anton Ficai, Denisa Ficai, Raluca Tutuianu, Ludmila Motelica, Vasile Adrian Surdu, Ovidiu-Cristian Oprea, Roxana Doina Truşcă, Irina Titorencu

**Affiliations:** 1 The National Research and Development Institute for Textiles and Leather, Lucretiu Patrascanu, 030508 Bucharest, Romania; alina_vladu_1995@yahoo.com; 2 Science and Engineering of Oxide Materials and Nanomaterials, Faculty of Chemical Engineering and Biotechnologies, National University of Science and Technology POLITEHNICA Bucharest, Gh. Polizu 1-7, 011061 Bucharest, Romania; anton.ficai@upb.ro (A.F.); motelica_ludmila@yahoo.com (L.M.); vasile.surdu@unitbv.ro (V.A.S.); truscaroxana@yahoo.com (R.D.T.); 3 National Center for Micro and Nanomaterials, National University of Science and Technology POLITEHNICA Bucharest, Splaiul Independentei 313, 060042 Bucharest, Romania; denisa.ficai@upb.ro (D.F.); ovidiu73@yahoo.com (O.-C.O.); 4 Division of Leather and Footwear Research Institute, Department of Collagen, National Research and Development Institute for Textiles and Leather, 93 Ion Minulescu Street, 031215 Bucharest, Romania; 5 Academy of Romanian Scientists, Ilfov Street 3, 050044 Bucharest, Romania; 6 Department of Inorganic Chemistry, Physical Chemistry and Electrochemistry, Faculty of Chemical Engineering and Biotechnologies, National University of Science and Technology POLITEHNICA Bucharest, Gh. Polizu 1-7, 011061 Bucharest, Romania; 7 Institute of Cellular Biology and Pathology “Nicolae Simionescu”, 8 B. P. Hasdeu Street, District 5, 050568 Bucharest, Romania; irina.titorencu@icbp.ro; 8 Research Center for Advanced Materials, Products and Processes, National University of Science and Technology POLITEHNICA Bucharest, 060042 Bucharest, Romania; 9 Department of Materials Science, Faculty of Materials Science and Engineering, Transilvania University of Braşov, Bv. Eroilor 29, 500036 Brasov, Romania

**Keywords:** bone graft, collagen, hydroxyapatite, doxorubicin, antitumoral, caffeic acid

## Abstract

The global burden of cancer continues to grow, with bone cancer—though rare—posing serious challenges in terms of treatment and post-surgical reconstruction. Autologous bone grafting remains the gold standard, yet limitations such as donor site morbidity drive the search for alternative solutions. Tissue engineering, combining biomaterials and therapeutic agents, offers promising avenues. This study focuses on the development of multifunctional scaffolds based on collagen and hydroxyapatite obtained by the freeze-drying technique and incorporating both synthetic (doxorubicin) and natural (caffeic acid) compounds for osteosarcoma treatment. These scaffolds aim to combine tumor inhibition with bone regeneration, addressing the dual need for local drug delivery and structural repair in bone cancer therapy. The characterization of these composite materials revealed that a spongious structure with interconnected pores and a homogeneous pore distribution, with pore sizes between 20 and 250 μm suitable for osteoblasts infiltration. The Fourier transform infrared (FTIR) spectroscopy and thermogravimetric analysis-differential scanning calorimetry (TG-DSC) and X-ray diffraction (XRD) analyses confirmed the formation of hydroxyapatite inside the collagen matrix. LDH and XTT assays confirmed that the antitumoral scaffolds possess great potential for osteosarcoma treatment, showing that after 3 days of culturing, the extracts containing doxorubicin-7A, both alone and in combination with caffeic acid-9A, significantly reduced the viability of cell lines to below 7% and 20%, respectively.

## 1. Introduction

In 2024, global statistics revealed a continued rise in cancer cases, with an estimated 20 million new diagnoses, while in 2022, about 9.7 million deaths caused by cancer were recorded. Bone cancer, while less common than other types, contributes to this global cancer burden. The report forecasts that overall cancer cases could reach 35 million annually by 2050, driven largely by aging populations and lifestyle factors like diet and smoking [1,2].

Bone cancer remains a significant health concern, affecting both children and adults, with a considerable impact on morbidity and mortality. Recent studies indicate that primary bone cancers, such as osteosarcoma, account for approximately 0.2% of all malignancies, with an annual incidence rate of about 3.4 cases per million people globally, predominantly affecting adolescents and young adults [3,4]. Survival rates for bone cancer have improved over the decades, yet the need for effective reconstructive options following tumor resection remains high [5].

Bone grafting is a widely utilized surgical technique for promoting bone regeneration in orthopedic procedures. All over the world, more than two million bone grafting procedures are performed each year, which makes it the second most common tissue transplantation after blood transfusion [6]. Autologous bone grafting is still considered the gold standard for bone tissue repair due to its histocompatibility and non-immunogenic nature, while also ensuring the essential properties needed for effective bone grafting. Moreover, it has been proven to enhance osteoinduction, osteogenesis, and osteoconduction [7]. Although bone grafting has multiple benefits, it also presents several drawbacks such as secondary damage, deformity, scarring, significant donor site injury, and morbidity and surgical risks, including bleeding, inflammation, and considerable costs [7,8]. Autografts are limited, and thus the use of the autografts is strongly limited and cannot be used for bone defects that require a larger volume of graft material [7]. Engineered (synthetic) grafts represent an emerging alternative to traditional bone grafts, being able to ensure an unlimited supply and removing the risk of disease transmission. However, various challenges and drawbacks have prevented bone tissue engineering from advancing to clinical trials. This approach focuses on facilitating functional bone regeneration through the combined use of biomaterials, cells, and factor therapy [9]. Jaisankar et al. functionalized single-walled carbon nanotubes (SWCNTs) with poly(methyl methacrylate) (PMMA) through controlled radical polymerization (CRP) by using SWCNT-Br as an initiator. The method produced SWCNT-g-PMMA with a low polydispersity index (PDI = 1.36), and gel permeation chromatography (GPC) demonstrated improved control without needing a sacrificial initiator. Various characterization techniques such as TGA, Raman spectroscopy, transmission electron microscopy (TEM), nuclear magnetic resonance (NMR), and X-ray photoelectron spectroscopy (XPS) confirmed successful functionalization, resulting in composites that are suitable as bone cement mimics [10]. As these biological scaffolds are drawing attention for bone repair, those with bioactivity alone cannot effectively treat tumors. Integrating therapeutic compounds within bioactive scaffolds to create bifunctional scaffolds that offer both therapeutic and reparative functions could be a promising future approach for bone tumor treatment [11,12]. Recent studies have shown that it is possible to embed therapeutic agents directly into bone-regenerative scaffolds, which can both eliminate tumors and promote bone tissue repair. For example, Chen et al. created a bortezomib-loaded nano-hydroxyapatite/alginate scaffold (BTZ/nHA@SA) that not only inhibited tumor growth but also improved bone healing in preclinical tests [13]. Likewise, a composite scaffold with Pt(IV) prodrug, polydopamine, and bioactive glass enabled combined photothermal chemotherapy, immunotherapy, and bone regeneration, showing strong anti-osteosarcoma effects and promoting bone formation [14]. Another method used a bionic bilayer scaffold combining croconaine dye-induced hyperthermia with a hydroxyapatite matrix, which effectively ablated tumors and repaired osteochondral tissue in an orthotopic osteosarcoma model [15]. These design approaches complement bifunctional scaffolds and support embedding therapeutic compounds into bioactive matrices for future bone tumor treatments.

Doxorubicin (DOX) is an anthracycline drug derived from the fungus *Streptomyces peucetius* and has powerful anti-cancer properties. It has been widely used in clinical settings to treat various cancers, such as bladder cancer, breast cancer, acute lymphocytic leukemia, and lymphoma, by exerting potent cytotoxic effects, particularly in rapidly proliferating cells [16,17]. The primary mechanisms behind its anticancer and cytotoxic effects are linked to its ability to intercalate between base pairs, thereby disrupting DNA metabolism and RNA synthesis. Additionally, it inhibits DNA replication and transcription and induces strand breaks by blocking the activity of the enzyme topoisomerase II [18,19]. DOX’s anticancer effects are further amplified by its quinone group, which facilitates redox cycling and generates reactive oxygen species (ROS), leading to cell damage and death. In addition to this, recent studies have uncovered other mechanisms of action, such as inducing senescence, autophagy, pyroptosis, and ferroptosis, while also modulating the anti-tumor immune response [19]. To enhance applicability and minimize chemoresistance and dosage-dependent toxicity, it is crucial to develop a drug delivery system (DDS) that ensures controlled, localized administration and sustained doxorubicin release. To achieve this, researchers have explored DOX integration with nanoparticles, liposomal formulations, and polymers [20,21,22]. Liu et al. investigated a calcium sulfate/hydroxyapatite (CaS/HA) carrier for localized, sustained doxorubicin (DOX) delivery to enhance osteosarcoma treatment. In vitro studies demonstrated controlled drug release, with 28% and 36% released over four weeks under physiological (pH 7.4) and acidic (pH 5) conditions, respectively, while in vivo release reached 63%. The released DOX remained effective against MG-63 and 143B osteosarcoma cells, comparable to free DOX. In xenograft models, CaS/HA-mediated DOX delivery significantly inhibited tumor growth by suppressing angiogenesis and cell proliferation, outperforming both systemic DOX administration and no treatment [23]. Kovrlija et al. successfully synthesized bioactive and biocompatible doxorubicin (DOX)-loaded octacalcium phosphate (OCP) particles in situ, with drug loadings ranging from 1 to 20 wt%. XRD analysis revealed that above 10 wt% DOX, OCP formation was inhibited, leading to the formation of low-crystalline α-TCP. In vitro drug release studies showed continuous DOX release over six weeks, with an initial burst in the first 24 h ranging from 15.9 to 33.5% at pH 7.4 and from 23.7 to 36.2% at pH 6.0. DOX-OCP effectively inhibited osteosarcoma cell lines (MG63, U2OS, and HOS), while MC3T3-E1 cells exhibited increased viability and proliferation over 3–7 days [22].

Polyphenols such as curcumin, resveratrol, epigallocatechin gallate, and caffeic acid represent another class of biological compounds that have garnered significant attention due to their potent antioxidant properties, which contribute to their therapeutic potential against cancer. These compounds effectively reduce oxidative stress associated with pathological conditions like cancer. Their ability to scavenge reactive oxygen species (ROS) and neutralize free radicals stems from their structural features, including aromatic rings, hydroxyl groups in various regions, and an extensively conjugated system [24,25]. Polyphenols can be combined with traditional cytostatic agents to potentiate their therapeutic effects against cancer cells while also protecting healthy cells from the harsh effects of antitumor drugs, thanks to their anti-inflammatory properties [25].

Caffeic acid (CA) or 3,4-dihydroxycinnamic acid is a polyphenolic secondary metabolite present in various plant tissues and dietary sources such as coffee, apples, and blueberries, as well as in propolis-based pharmaceuticals. It possesses diverse biological properties, including antioxidant, anticancer, antiviral, anti-HIV, immunoregulatory, and anti-inflammatory effects. Importantly, caffeic acid influences apoptosis and autophagy in malignant cells, thereby affecting their growth and survival [26,27,28]. It has shown effectiveness against several cancer types, including head and neck carcinoma, MCF-7 breast cancer, osteosarcoma (MG-63) cells, multiple forms of myeloma (MM.1R, RPMI8226, and U266), and leukemia (K562) [29]. Apoptosis is a crucial mechanism that takes place under physiological conditions and during normal developmental processes. It occurs through two distinct pathways: the extrinsic and intrinsic pathways. The extrinsic pathway is triggered by death receptors on the cell membrane, such as those belonging to the tumor necrosis factor (TNF) receptor superfamily. In contrast, the intrinsic pathway is activated by factors like DNA damage and growth factor deprivation, leading to initiation of the caspase cascade [30]. Treatment with caffeic acid (0–200 μM) promotes apoptosis and induces cell cycle arrest in melanoma cells while also suppressing colony formation. Expression analysis revealed an upregulation of caspase-1, -3, and -8 following CA exposure, emphasizing its anti-melanoma effects [31]. Additionally, CA has been demonstrated to enhance the expression of p53, an important tumor suppressor protein which plays a key role in cancer prevention; p53 activation can lead to cell cycle arrest and apoptosis [32]. Su et al. developed a 3D-printed scaffold (CCNACA) incorporating carbon dots-curcumin nanoparticles (CCNPs), sodium alginate, nanoclay, and caffeic acid-grafted chitosan for localized drug delivery and real-time visualization of drug release. In vitro studies demonstrated that CCNACA scaffolds achieved a 73.77 ± 1.68% tumor inhibition rate against MCF-7 breast cancer cells over 14 days. Additionally, the scaffolds exhibited sustained antibacterial activity against *Escherichia coli* and *Staphylococcus aureus*. In vivo experiments further revealed that CCNACA scaffolds effectively suppressed residual cancer cells, reduced inflammation, promoted angiogenesis, and facilitated tissue repair following surgical implantation [33]. Researchers encapsulated quercetin and caffeic acid phenethyl ester (CAPE) within poly(lactic-co-glycolic acid) (PLGA) nanoparticles, resulting in enhanced cytotoxicity against HT-29 colon cancer cells. This formulation promoted higher expression of apoptotic markers and demonstrated superior antitumor efficacy compared with the free compounds [34]. Additionally, the studies that compared CA with its derivative, caffeic acid phenethyl ester (CAPE), showed that CAPE demonstrates higher cytotoxicity and selectivity against osteosarcoma cells. It more effectively reduces tumor cell viability while preserving normal osteoblasts, indicating that structural modifications of CA may increase its anticancer potential [35]. Zhu et al. revealed that epigallocatechin gallate (ECGC) is able to inhibit osteosarcoma by upregulating miR-1, causing c-MET suppression. This results in reduced proliferation, increased apoptosis, and enhanced tumor suppression, especially when mixed with the c-MET inhibitor crizotinib [36].

The response of cancer cells to therapy can be boosted using combination cancer therapy. The co-loading of caffeic acid (CA) with 5-fluorouracil (5-FU) enhances the physicochemical properties of 5-FU, improving its solubility and tissue permeability. This synergistic interaction enhances 5-FU’s anticancer efficacy, as evidenced by a combination index (CI) of less than one [37]. Su et al. demonstrated that curcumin (CUR) effectively reduces chemoresistance in CRC cells by inducing apoptosis in IRI-resistant cells. CUR significantly modulates CSC identification marker expression and enhances pro-apoptotic Bax while suppressing anti-apoptotic Bcl-2 [38,39]. The combination of cisplatin and tea polyphenols, particularly EGCG, enhances cell cycle arrest, antioxidant activity, and apoptosis while reducing drug resistance and toxicity. This synergy improves chemosensitization in ovarian cancer and inhibits the growth of various cancer cells, including MCF-7 breast cancer cells and A549 lung cancer cells [40]. Another study emphasized that low concentrations of catechins (EGCG, EGC, and ECG) selectively induce cytotoxicity in ERα-negative breast cancer cells. While ineffective alone in T47D cells, EGCG showed mild cytotoxicity in MCF-7 cells. In MDA-MB-231 cells, EGCG exhibited higher cytotoxicity than tamoxifen, and their combination produced a synergistic effect. These findings highlight the potential of catechins, particularly EGCG, in enhancing breast cancer treatment [41]. Considering the proven potential of combination therapy in cancer treatment, the focus of this study is to evaluate the benefits of designing scaffolds which contain a mixture of natural and synthetic compounds, namely caffeic acid and doxorubicin, which can be used in osteosarcoma treatment and bone tissue engineering.

## 2. Materials and Methods

### 2.1. Materials

For the synthesis of collagen/hydroxyapatite (Coll/HAp) scaffolds, type I collagen (Coll) gel was utilized, obtained from bovine skin through a method developed by the Collagen Research Department at INCDTP—Leather and Footwear Research Institute, having a collagen concentration of 1.67% (*w/v*). Hydroxyapatite was synthesized in situ from di-ammonium hydrogen phosphate ((NH_4_)_2_HPO_4_) with a purity of ≥97% from Roth (Karlsruhe, Germany) and calcium hydroxide (Ca(OH)_2_) with a purity of ≥96% from Fluka (Buchs, Switzerland). Additionally, sodium hydroxide (NaOH) with a purity of 99.45% was sourced from Lach-Ner (Neratovice, Czech Republic). The samples were loaded with doxorubicin hydrochloride (DOX) at ≥98% purity and caffeic acid (CA) at ≥98% purity, both of which were purchased from Sigma Aldrich (St. Louis, MA, USA). The crosslinking agent, genipin (GP) at ≥98% purity, was also obtained from Sigma Aldrich (St. Louis, MA, USA). All reagents were used without further purification. For cell culture, human osteosarcoma cell line MG63 (ATCC CRL1427) was cultured in Dulbecco’s Modified Eagle’s Medium-DMEM supplemented with 1% non-essential amino acids, both from Sigma Aldrich, 10% fetal calf serum (FBS) from Thermo Fisher Scientific (Waltham, MA, USA), and 1% penicillin, streptomycin, and neomycin (all from Sigma Aldrich) at 37 °C with 5% CO_2_. Human bone marrow-derived mesenchymal stem cells (BMSCs) were previously obtained and characterized in the Cell and Tissue Engineering Laboratory of the Institute of Cellular Biology and Pathology “Nicolae Simionescu” in Bucharest, Romania [42].

### 2.2. Preparation of Composite Materials

The samples were obtained by freeze-drying according to previous studies and loaded with two different active compounds [43,44]. The Coll/HAp composite materials at a 1:1 ratio were obtained by mixing the collagen gel with calcium and phosphate precursors while maintaining a basic pH of 9. After rinsing the resulting gel with distilled water, the active compounds were introduced. Doxorubicin hydrochloride was dissolved in distilled water, and caffeic acid was dissolved in ethanol. Then, they were added to the composite gel to obtain the compositions presented in Table 1.

**Figure 1:**
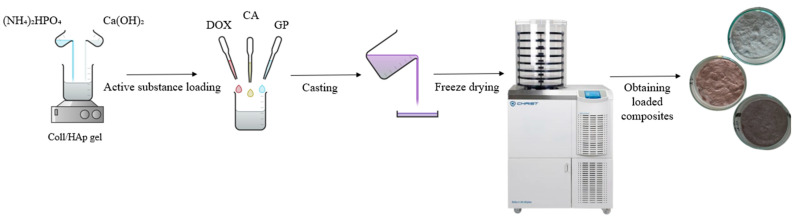
Schematic representation of the preparation of antitumoral composite materials.

The gel was crosslinked using genipin and cast into glass Petri dishes (5.2 cm in diameter, 1 cm high). To obtain spongy matrices, the samples underwent freeze-drying using a Delta LSC 2-24 lyophilizer (Martin Christ, Osterode am Harz, Germany). The process began with pre-cooling the lyophilizer shelves to −40 °C, where the samples were held for 1.5 h, followed by a 48-h freeze-drying cycle. Figure 1 presents the schematic process of obtaining the Coll/Hap-loaded sponge composites.

The obtained composite materials were characterized using adequate analytical techniques as follows.

### 2.3. Characterization Techniques

#### 2.3.1. Water Absorption

To evaluate water absorption, small composite samples (0.0072–0.0141 g) were initially weighed dry and then immersed in 3 mL of water at 25 °C. Samples were reweighed at predetermined intervals (1, 2, and 4 h; 1, 2, and 3 days) to determine water uptake using the following formula:

(1) $$\mathrm{Water}\;\mathrm{uptake}\;(\%)\;=\;\frac{Wt-Wd}{Wd}\;100$$

where *Wt* is the weight at time *t* and *Wd* is the initial dry weight. Values are reported as the mean ± standard deviation from triplicate experiments [45].

#### 2.3.2. Enzymatic Degradation

The degradation behavior of the Coll/HAp composites was assessed by immersing the hydrated samples in a collagenase solution (1 μg/mL). The weight loss over time (one week) was monitored using the following formula:

(2) $$\mathrm{Weight}\;\mathrm{loss}\;(\%)\;=\;\frac{Wi-Wt}{Wt}\;100$$

where *Wi* is the initial weight and *Wt* is the weight at each time point. The results represent the mean ± standard deviation from three replicates [45].

#### 2.3.3. Scanning Electron Microscopy (SEM)

The surface morphology and pore structure of the composites were examined via scanning electron microscopy (SEM) using a Quanta Inspect F50 instrument (FEI Company, Eindhoven, The Netherlands). The SEM, equipped with a field emission gun and energy-dispersive X-ray spectrometer (resolution: 1.2 nm; 133 eV MnK), provided high-resolution images for analysis. The porosity was quantified using ImageJ (Java 1.54g).

#### 2.3.4. FTIR Spectroscopy

FTIR spectroscopy (Nicolet iS50, Thermo Fisher Scientific) was utilized to identify functional groups in the 400–4000-cm^−1^ spectral range. Further spatial characterization of the component distribution was performed using FTIR microscopy with a Nicolet iN10 MX system.

#### 2.3.5. Thermogravimetric Analysis (TGA)

Thermal behavior was investigated using an STA 449C F3 TG-DSC (Netzsch, Gerätebau GmbH, Selb, Germany) system under dynamic airflow (50 mL/min) from 20 to 900 °C. Evolved gases were continuously analyzed via a Bruker FTIR Tensor 27 spectrometer (Bruker Co., Ettlingen, Germany) equipped with a temperature-controlled gas cell.

#### 2.3.6. X-Ray Diffraction (XRD)

X-ray diffraction (XRD) patterns were obtained utilizing an Empyrean X-ray diffractometer (Malvern PANalytical, Cedar Park, TX, USA) equipped with Ni-filtered CuKα radiation (λ = 1.5418 Å). Data acquisition was performed over a 2θ range spanning from 10° to 80°, with a step size of 0.026° and a counting duration of 255 s per step. Phase identification was carried out using HighScore Plus software (version 3.0.e) in conjunction with the COD 2024 database. Crystallite size analysis was conducted by employing the Rietveld refinement method, which included the application of a polynomial function for background modeling, a pseudo-Voigt function for peak shape analysis, and the Caglioti function to account for peak broadening. The crystallinity percentage [%] of the samples was determined within the HighScorePlus 3.0.e software by calculating the intensity ratio of the diffraction peaks relative to the total measured intensity.

#### 2.3.7. Lactase Dehydrogenase Enzyme (LDH) and XTT Assays

Sample extracts were prepared by incubating the constructs in complete growth medium for 8 h at 37 °C under agitation. The supernatant was sterilized by filtration using 0.2-µm syringe filters and stored at 4 °C. Cells were seeded in 96-well plates at a density of 10^4^/cm^2^, and after 24 h, they were incubated in triplicate with 100% extracts; complete medium without the test extract was considered a control. LDH activity was determined using a cytotoxicity detection kit (Roche, cat. no. 4744934001) according to the manufacturer’s instructions. Briefly, 100 µL of a freshly prepared reaction mix was added to each well, followed by 50 µL of stop solution after 30 min of incubation in the absence of light. The absorbance was measured at 490 nm versus 600 nm using a TECAN spectrophotometer. The positive control for the LDH release was obtained by adding 5 µL/well of Lysis buffer from the kit. The negative control was the cells cultured in complete growth medium without extracts. The results were expressed as the fold LDH release by dividing the values for the test samples by the ones in the negative control group. The cell viability was also assessed by measuring their metabolic activity using the XTT test (Invitrogen). For this, the cells were washed with warm PBS and incubated with the 100 µL/well of XTT working solution as per the manufacturer’s protocol. After 2 h at 37 °C and 5% CO_2_, the absorbance was read at 450 nm versus 690 nm using a TECAN spectrophotometer. The control for these experiments was cells cultured in complete growth medium without extracts. The results were expressed as percentages relative to the control.

#### 2.3.8. Statistical Analysis

Statistical analysis of the data was performed using GraphPad Prism 9.1.0 software (San Diego, CA, USA). Two-way ANOVA was used for the comparison of the experimental groups, and *p* values less than 0.05 were considered statistically significant.

## 3. Results and Discussion

### 3.1. Water Uptake Capacity of Loaded Coll/HAp Sponges

**Figure 2:**
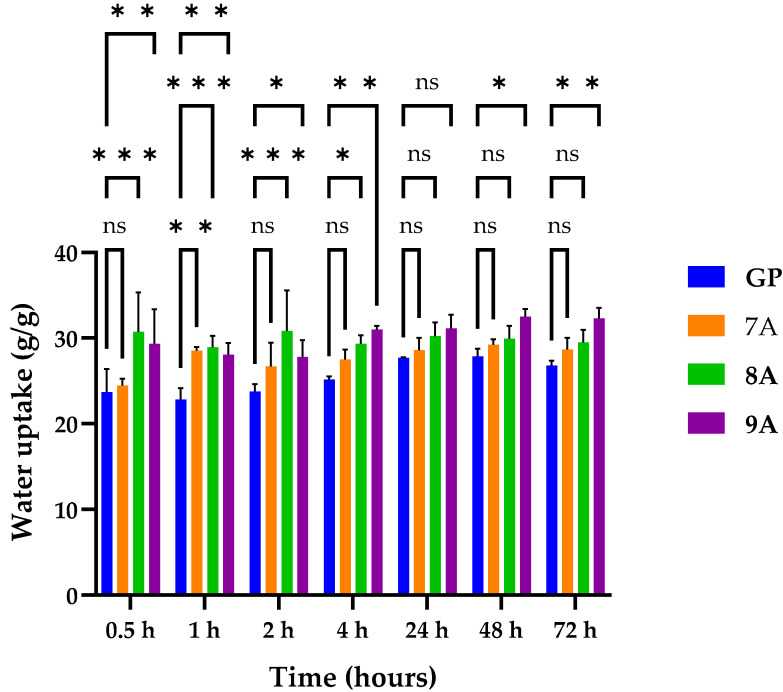
Water uptake for 7A, 8A, 9A, and GP matrices. Results were not statistically significant (ns) for *p* value >0.05, and results were statistically significant for *p* < 0.05 (*), *p*  <  0.01 (**), and *p*  <  0.001 (***).

Figure 2 illustrates a diagram of the water uptake capacity obtained for the loaded Coll/HAp composite materials. It shows that all samples absorbed a consistent amount of water (~23–32 g/g).

The samples retained a significant amount of water shortly after immersion and in the following hours, with most of them stabilizing after 24 h, reaching equilibrium. The diagram does not show significant variation in the water uptake capacity, although sample 9A exhibited slightly increased water absorption, probably as a result of the enhanced hydrophilicity conferred by the two components: doxorubicin and caffeic acid. The GP sample absorbed a lower amount of water than the other samples, most likely because of the hydrophobic nature of genipin and because genipin and hydroxyapatite may have increased the scaffold density. In the first four hours, the water uptake values of the 8A and 9A samples were statistically significant compared with the water uptake value of the GP sample (* *p* < 0.05, **** *p* < 0.001), while the water uptake value of the 7A matrix was statistically significant only after one hour after the beginning of the measurements. After 24 h, none of the formulations were statistically significant due to reaching the equilibrium state. This trend was partially conserved for 48 and 72 h, except for the 9A sample, whose water uptake increase was significant compared with GP (* *p* < 0.05, ** *p* <  0.01).

### 3.2. Enzymatic Degradation of Loaded Coll/HAp Sponges

The degradation of loaded Coll/HAp composites was assessed by immersing the samples in a collagenase solution with a 1 μg/mL concentration, an enzyme that catalyzes the breakdown of collagen into amino acids.

**Figure 3:**
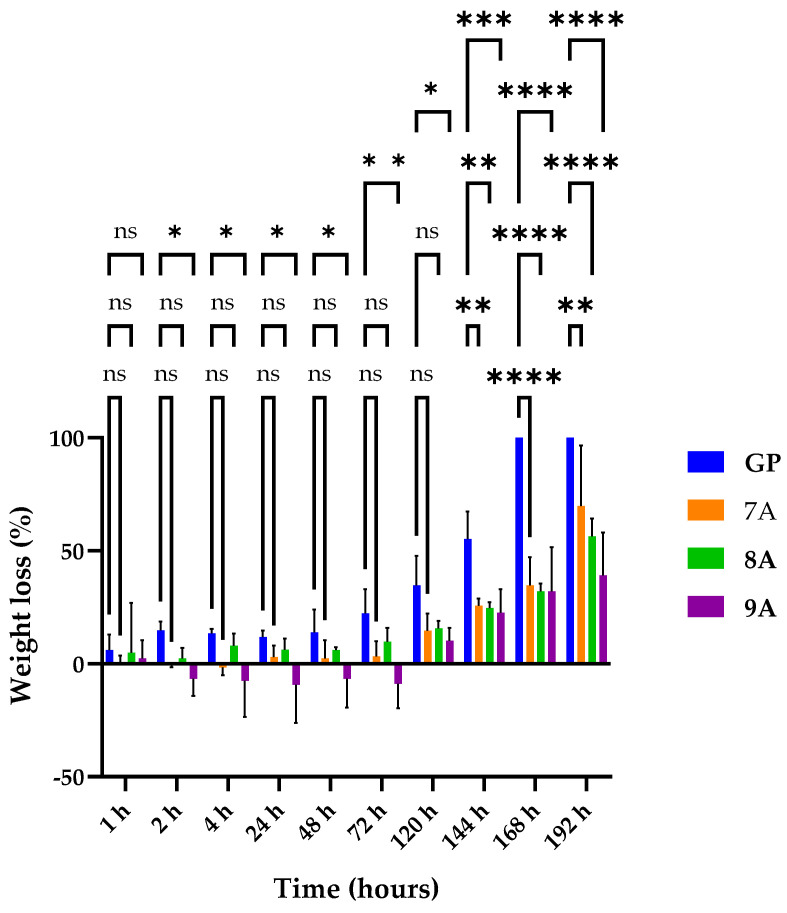
Enzymatic degradation for 7A, 8A, 9A, and GP matrices. Results are not statistically significant (ns) for *p* > 0.05. Results are statistically significant for * *p* < 0.05, ** *p*  <  0.01, *** *p*  <  0.001, and **** *p*  <  0.0001.

Figure 3 illustrates the enzymatic degradation behavior of the obtained samples. Complete degradation was observed exclusively for the GP sample, occurring after 6 days. Negative values were observed for 7A and 9A, with both samples consisting of doxorubicin. This can likely happen due to the doxorubicin inhibition of collagenase [46]. The 9A sample started degrading later, with the inhibition of doxorubicin to collagenase being potentiated by the presence of caffeic acid. The loaded samples had a lower degradation rate compared with the control, suggesting that they are more stable and resistant to degradation. The slower enzymatic degradation observed in sample 8A compared with sample 7A could be attributed to the presence of caffeic acid, which may exert a mild inhibitory effect on collagenase activity and promote structural stabilization through hydrogen bonding or weak crosslinking. In contrast, the doxorubicin in sample 7A did not appear to confer similar enzymatic resistance. Among the tested formulations, sample 9A exhibited the lowest enzymatic degradation after 7 days (~40% mass loss), suggesting a synergistic stabilizing effect from both caffeic acid and doxorubicin. These components may contribute to reduced enzymatic accessibility and partial inhibition of collagenase activity. In the first hour, none of the matrices registered a statistically significant modification of weight loss compared with GP. In the time intervals of 2 h and 120 h, sample 9A recorded a significant decrease in weight loss compared with GP (* *p* < 0.05, ** *p* <  0.01), while the changes in weight loss for the other matrices were insignificant. After 144 h, all three matrices had significantly less weight loss than that of GP (** *p* <  0.01, **** *p* <  0.0001), suggesting that the addition of the other components (doxorubicin and caffeic acid) increased the structural resistance of the matrices compared with GP, which after 168 h was completely digested by collagenase.

### 3.3. Scanning Electron Microscopy (SEM) and Energy-Dispersive X-Ray Spectroscopy (EDX) Characterization

**Figure 4:**
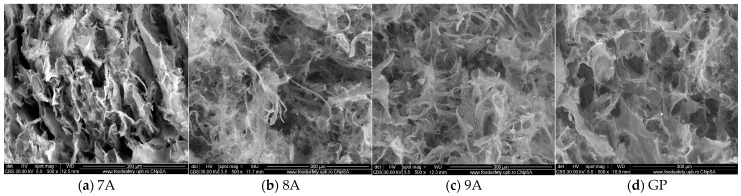
SEM micrographs of loaded composite scaffolds (x500): (**a**) 7A, (**b**) 8A, (**c**) 9A, and (**d**) GP.

The microstructure of the collagen/hydroxyapatite composite materials loaded with doxorubicin and caffeic acid was observed via SEM after freeze-drying (Figure 4). The samples exhibited similar microstructures, with a sponge-like structure and increased porosity. Freeze-drying significantly enhanced the scaffold’s morphological characteristics, leading to the formation of an open architecture with interconnected pores and a homogeneous pore distribution. The typical platelet-like structure of collagen could also be observed, connected by additional platelets and fibers. The size of the pores was between 20 and 250 μm, which is expected to facilitate osteoblast migration and promote the diffusion of nutrients and metabolic byproducts [47]. The inorganic phase was homogeneously distributed inside the collagen matrix and of a nanometric size.

**Figure 5:**
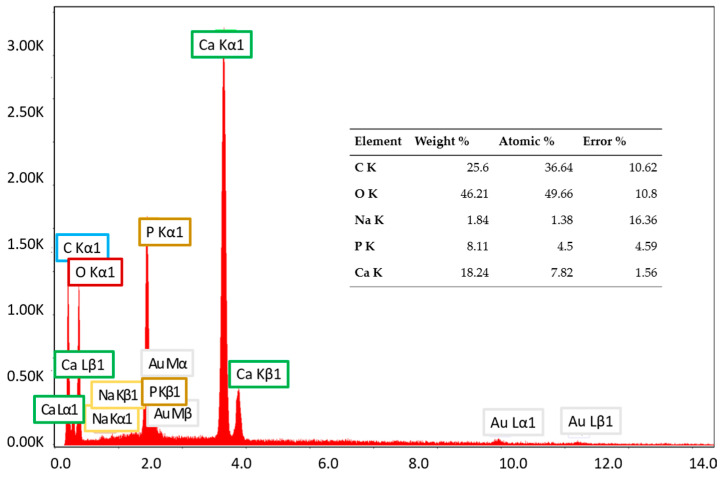
EDX spectrum and elemental composition of GP sample.

Scanning electron microscopy coupled with energy-dispersive X-ray analysis (SEM-EDX) was employed to determine the elemental compositions of the samples. The analysis revealed the presence of calcium, phosphorus, oxygen, carbon, and sodium (Figure 5).

Calcium, phosphorus, and oxygen indicate the presence of hydroxyapatite. Although the Ca/P ratio was 1.74, higher than 1.67, which is the value specific to stoichiometric hydroxyapatite, the inorganic phase can be attributed to non-stoichiometric hydroxyapatite of biological or synthetic origin. Additionally, the detection of sodium is attributed to residual NaCl from the collagen synthesis process, which was present in non-toxic amounts. The low sodium content is most likely due to its weak interaction with the collagen matrix, leading to its removal during washing or dialysis. Since sodium ions are not covalently bonded and are highly soluble, they were not substantially retained in the final product. The lack of chlorine in the material indicates that Cl^−^ was effectively removed during purification. While collagen is typically extracted using saline solutions, any remaining Cl^−^ seems to have been washed away. However, if trace amounts of Cl were detected, then they could have originated from the saline used in collagen preparation.

### 3.4. FTIR Spectroscopy and Microscopy

**Figure 6:**
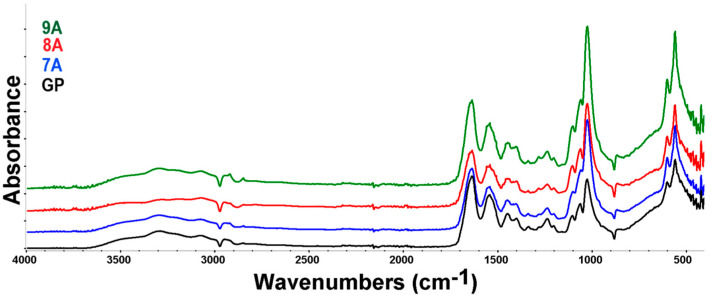
FTIR spectra for samples 7A, 8A, 9A, and GP.

FTIR spectroscopy was further used to identify the main characteristic absorption bands of the organic and inorganic phases present in the samples. By analyzing the FTIR spectra of the loaded composites (Figure 6), the characteristic absorption bands of collagen and hydroxyapatite could be clearly distinguished, despite some overlap occurring in certain spectral regions.

**Figure 7:**
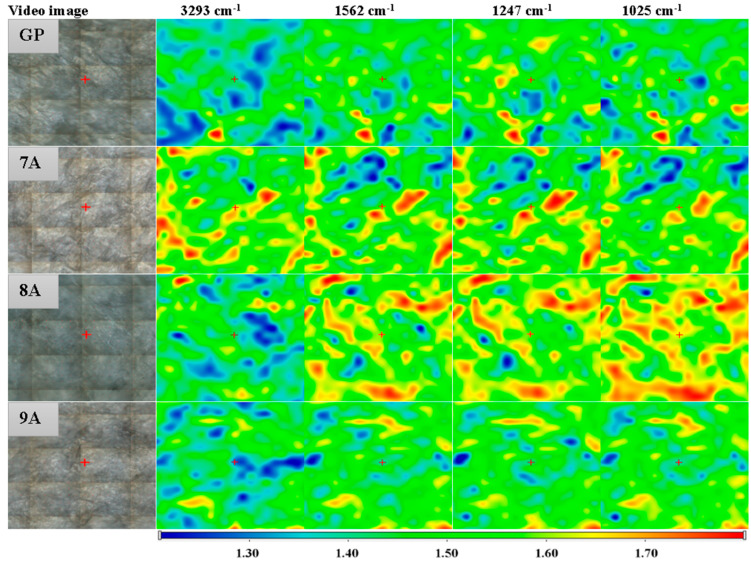
The FTIR maps at 3293 cm^−1^, 1562 cm^−1^, 1247 cm^−1^, and 1025 cm^−1^ for the 7A, 8A, 9A and GP samples. Red areas indicate the highest absorbance, while blue areas correspond to the lowest absorbance.

Thus, the presence of collagen can be confirmed by the absorption bands observed at the wavenumbers of 3294 cm^−1^, specific to N-H stretching, 1636 cm^−1^ (amide I-α helix, C=O stretching), 1541 cm^−1^ (amide II, N-H bending and C-N stretching), 1448 cm^−1^ (N-H bending), and 1237 cm^−1^, characteristic of amide III corresponding to N–H bending vibrations, coupled with C–H and C–N stretching modes [48]. The hydroxyapatite bands could be found at 1025, 1103, 559, and 601, which are assigned to the ${PO}_{4}^{3-}$ group and associated with symmetric bending and stretching vibrations [49]. Overlapping of the hydroxyapatite and collagen absorption bands occurs in the 3100–3400 cm^−1^ region, where hydroxyapatite’s O-H stretching can be found. Also, in the 1400–1636 cm^−1^ spectral region, the specific carbonyl stretching bands of hydroxyapatite overlap with those of collagen [50]. Minor shifts in the characteristic absorption bands of collagen and hydroxyapatite were observed. These shifts suggest potential molecular interactions—such as hydrogen bonding or electrostatic interactions—between the organic matrix (collagen, doxorubicin, genipin, and caffeic acid) and the inorganic phase (hydroxyapatite), indicative of composite integration at the molecular level. The presence of doxorubicin, caffeic acid, and genipin cannot be confirmed with certainty based on the analyzed FTIR spectrum, due to the small quantities that were used and significant overlap of its characteristic bands with those of collagen and other components. FTIR microscopy can offer information on the homogeneity of the polymer blend in the composite films. The chosen wavenumbers were specific to water, collagen, and hydroxyapatite. Figure 7 presents the FTIR maps for the GP sample at 3293 cm^−1^, 1562 cm^−1^, 1247 cm^−1^, and 1025 cm^−1^.

The FTIR maps recorded for these specific wavenumbers are similar, indicating that the films were homogeneous, with only minor variation related to the humidity (3293 cm^−1^), while COLL and HA were homogeneously distributed well. The slightly different distribution of the adsorbed humidity may be associated with the slightly heterogeneous adsorption of the DOX/CA.

### 3.5. TG-DSC Analysis

**Figure 8:**
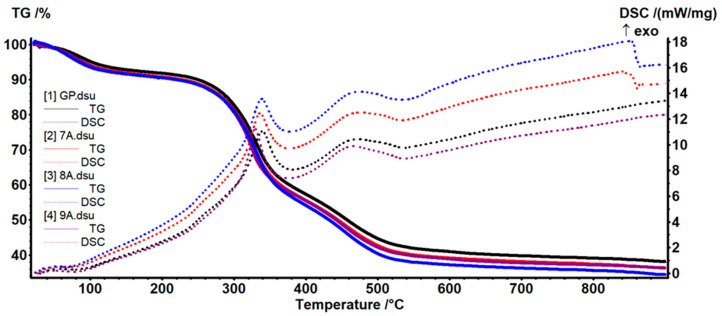
TG-DSC curves for samples 7A, 8A, 9A, and GP.

TG-DSC analysis was used to investigate the behavior of materials as a function of the temperature, and the curves are shown in Figure 8.

According to Table 2, the samples were losing ~8–9% of their initial mass up to 200 °C in a dehydration process.

The weak endothermic effect on the DSC curves had a minimum between 64 and 75 °C, supporting the hypothesis of water elimination from the fibrillary network of collagen [51]. The oxidative and degradative processes started after 200 °C and up to 540 °C, with the samples losing ~50–52% of their initial mass. This mass loss step was the result of at least two separate reactions, as indicated by the peaks of the DSC curves. The exothermic effects presented a maximum at 329–339 °C due to partial oxidation of the organic compounds, while the second maximum was at ~465–475 °C and could be attributed to the oxidation of the residual carbonaceous mass [52]. After 540 °C a small mass loss of ~4% was recorded, which could be assigned to the loss of –OH moieties from HAp and densification of the inorganic residue [43]. The residual mass was ~34–38%, with a lower value when doxorubicin or caffeic acid was added. A similar effect could be observed at the second mass loss step between 200 and 540 °C, when the oxidation of the organic molecules takes place, with the mass loss being slightly larger when additional organic compounds were added (doxorubicin or caffeic acid).

### 3.6. X-Ray Diffraction (XRD)

**Figure 9:**
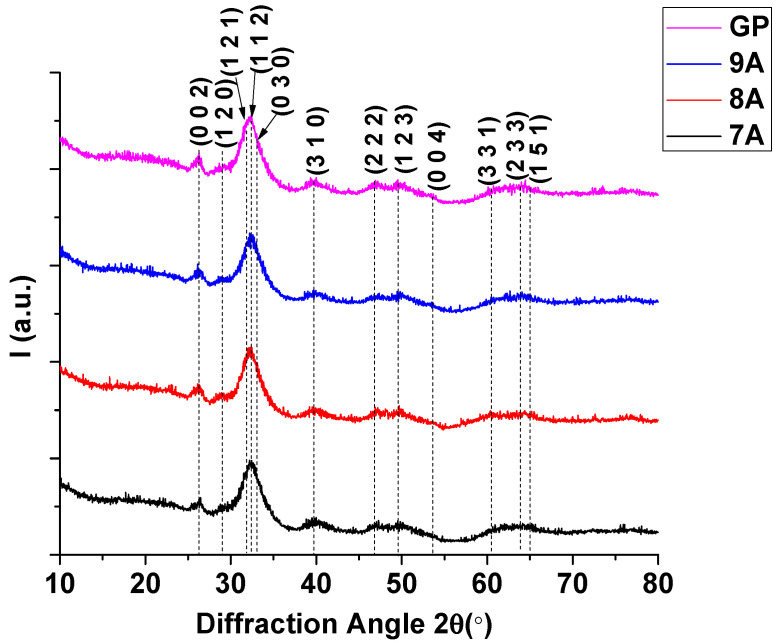
XRD patterns of antitumoral composites: 7A, 8A, 9A, and GP.

Diffractograms were obtained in order to confirm the in situ formation of hydroxyapatite (Figure 9).

The diffractograms obtained for the antitumoral composites included the diffraction peaks of the main planes of hydroxyapatite, namely (002) at 2θ = 25.88°, (120) at 2θ = 28.92°, (121) at 2θ = 31.76°, (112) at 2θ = 32.19°, (030) at 2θ = 32.89°, and (222) at 2θ = 46.69°, with a hexagonal structure. Phase identification was achieved using the COD 2024 database (COD # 96-901-1093) [53]. The X-ray diffraction pattern closely resembled that of natural bone, exhibiting broadened peaks at positions characteristic of hydroxyapatite. The peak broadening is indicative of nanoscale hydroxyapatite crystallites, suggesting the formation of a nanostructured phase. The crystallite size determined by the Rietveld refinement method was ~3 nm for each analyzed sample. This nanometric size is essential for the bioactive properties of bone. The crystallinity of the samples was ~30–31%, emphasizing a more amorphous biomimetic structure similar to that of natural bone, which is known to enhance bioactivity and resorbability, thereby promoting osteointegration and bone tissue regeneration [54].

### 3.7. Biological Assessment

**Figure 10:**
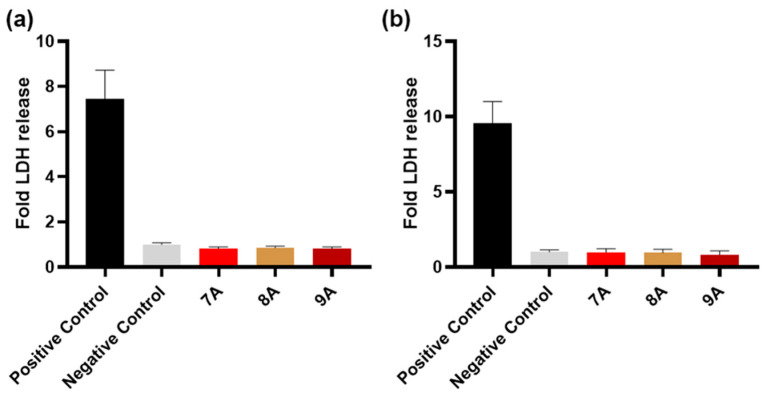
Cytotoxicity effect on MG63 (**a**) and BMSCs (**b**) after 24 h of exposure to the tested samples’ extracts, evaluated via the release of LDH enzyme. Complete growth media was used as a negative control, and lysed cells were used as a positive control. Results are expressed as mean ± standard deviation (n ≥ 3, independent samples).

After 24 h of exposure, both the MG63 and BMSC cell lines showed no significant release of LDH compared with the negative control, the cytotoxicity levels for all three samples’ extracts being similar to the ones found for the cells maintained in complete growth medium (Figure 10).

**Figure 11:**
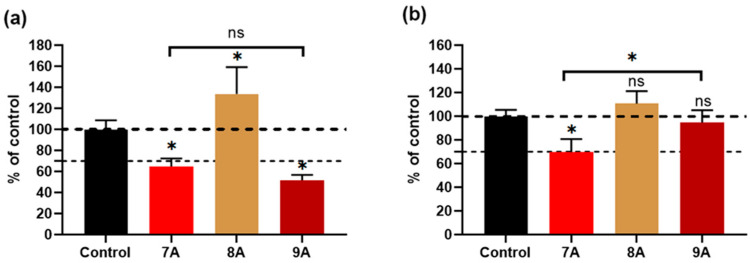
Cellular viability of MG63 (**a**) and BMSCs (**b**) cultured with 100% extracts from each sample, for 1 day. Complete growth media was used as a control. The dashed line indicates the 70% cut-off for a toxic effect. Results are expressed as mean ± standard deviation (n ≥ 3, independent samples, * *p* < 0.05).

Also, 24 h after extract incubation, the MG63 as well as the BMSC cell viability for the 7A sample was below the 70% threshold, while for 8A it was approximately 33% and 10% above that of the control, respectively, as seen in Figure 11. For sample 9A, the osteosarcoma cells showed a viability below the one for sample 7A (~51%) but without statistical significance (Figure 11a). In the case of the BMSCs, the 9A extract induced higher viability (~94% of the control) compared with 7A (~69%).

**Figure 12:**
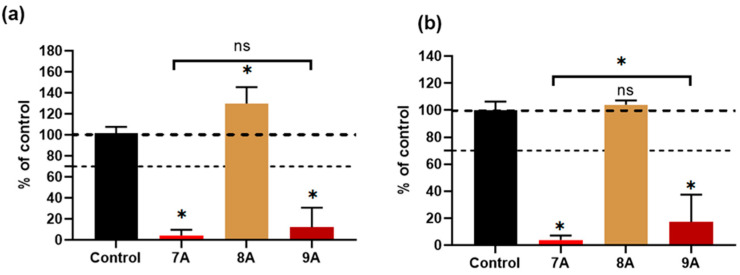
Cellular viability of MG63 (**a**) and BMSCs (**b**) cultured with 100% extracts from each sample for 3 days. Results are expressed as mean ± standard deviation (n ≥ 3; independent samples; * *p* < 0.05).

Figure 12 shows that after 3 days of culturing, the samples’ extracts containing DOX- 7A as well as those combined with CA–9A induced a significant reduction in the viability of both cell lines (below 7% and 20%, respectively). Also, for the BMSCs, their cultivation in the 9A extract was associated with higher viability, being ~17% compared with the 7A sample (~4%). The 8A extract maintained its effect observed 24 h post-incubation, with a viability above the 100% control value in the case of MG63 and BMSCs.

The obtained data on the MG63 osteosarcoma cell line show that both the 7A and 9A extracts inhibited malignant cell proliferation over time. Furthermore, for the bone marrow-derived mesenchymal stem cells (BMSCs), the cytotoxic effect seemed to be attenuated, especially after initial exposure, by the addition of CA, suggesting a possible protective action for this type of cell but not for the osteosarcoma cells, in the case of their exposure to the chemotherapeutic agent.

The combined action of doxorubicin and caffeic acid has been previously studied for lung cancer, with promising results both in vitro and in vivo, suggesting that these effects are mediated through the MAPK pathway [55]. Regarding MG63 cells, it is known that short exposure (up to 12 h) to caffeic acid can trigger both the intrinsic and extrinsic apoptosis pathways in this cell line [56]. On the other hand, stem cells are known to have a robust antioxidant defense system, especially based on high levels of total gluthatione [57] which could make them more resistant to the oxidative stress induced by doxorubicin. Furthermore, certain polyphenols were able to protect BMSCs against oxidative stress [58], and caffeic acid priming induced a lower level of ROS [59]. Therefore, the selective response of BMSCs revealed by our in vitro results could be explained by these cells’ resistance mechanisms being sustained by the action of caffeic acid.

In the present study, the results of the in vitro cytotoxicity and cell proliferation assays indicate the composite scaffolds containing both doxorubicin and caffeic acid (sample 9A) as suitable candidates for osteosarcoma inhibition. However, further validation via dose–response and mechanistic studies are needed to assess the potential for oncological and regenerative applications.

## 4. Discussion

Compared with earlier collagen–hydroxyapatite systems, our composite scaffolds feature a dual-agent combination of doxorubicin and caffeic acid, which has not been simultaneously studied for both bone cancer suppression and tissue regeneration. Unlike studies that only examined DOX or natural compounds individually, our system leverages the synergistic effect of CA, which seems to selectively offer protection to BMSCs. Table 3 highlights the main features of our scaffold (9A) and compares it with other scaffolds in the literature that also include chemotherapeutic agents for osteosarcoma treatment.

Compared with Rong et al. [60], our scaffolds have similar pore sizes (20–250 µm) but offer better selectivity, effectively targeting MG63 osteosarcoma cells and providing partial protection to BMSCs thanks to caffeic acid. While Rong’s system emphasizes long-term drug release, our materials show improved enzymatic stability and specific cellular responses. Unlike oriented nanofiber systems (e.g., those from Lu et al.) [61], which require complex fabrication and surface modifications for bioactivity, our materials maintain a biomimetic nanostructure and are easily produced through freeze-drying. Also, unlike Wang et al.’s 3D-printed scaffolds tuned for porosity and drug control [62], our freeze-dried matrices provide a simpler manufacturing process, achieving selective cytotoxicity and stability without complex structural patterning.

Furthermore, our 9A scaffold, loaded with DOX and CA, showed a synergistic biological effect—selectively killing cancer cells while supporting normal stem cell health—that has not been reported for basic collagen-based matrices. These features underscore the novelty of our approach in combining composition and biological specificity, offering a promising alternative for localized bone cancer treatment with regenerative benefits.

## 5. Conclusions

This paper focused on the development and evaluation of composite scaffolds made of collagen and hydroxyapatite loaded with doxorubicin and caffeic acid as potential multifunctional bone grafts used in cancer therapy. The composite materials were obtained using a freeze-drying method, and the experimental results indicate that the samples possessed the fundamental properties required for their application in bone tissue engineering and cancer therapy. The composite scaffolds exhibited interconnected pores ranging from 20 to 250 μm in diameter and a uniform pore distribution with homogeneous dispersion of nanometric hydroxyapatite particles. The open porous structure is expected to enhance osteoblast infiltration and nutrient diffusion. XRD analysis confirmed the in situ formation of hydroxyapatite, with the diffraction peaks matching a hexagonal HA (e.g., 2θ = 25.88°, 31.76°, 46.69°). The crystallite size was ~3 nm, indicating a nanostructured phase. The sample crystallinity was ~30–31%, suggesting a partially amorphous biomimetic structure favorable for osteointegration. After 3 days, the 7A and 9A extracts reduced MG63 cell viability to below 7% and 20%, respectively, while 9A maintained higher BMSC viability (~17%) compared with 7A (~4%), indicating that caffeic acid may offer selective protection to stem cells. This study illustrates the potential of collagen–hydroxyapatite composite scaffolds loaded with DOX and CA as targeted combination therapy for osteosarcoma. The dual benefits—anticancer activity and cytocompatibility—underscore these scaffolds’ promise for oncological treatment and bone regeneration. The selectively protective effect of CA on BMSCs likely results from its antioxidant properties and compatibility with stem cell defense mechanisms. These results imply that CA can improve the therapeutic index of DOX in a local delivery system. Future research will include in vivo assessment of scaffold performance, deeper exploration of DOX–CA interactions within the tumor microenvironment, and optimization of drug loading and release. If confirmed, then this composite system could become a minimally invasive therapeutic option after tumor resection in orthopedic oncology.

## Figures and Tables

**Table 1:** Codification and composition of loaded Coll/HAp composite materials.

Sample Code	Coll/HAp 1:1 Mineralized Gel	DOX	CA	GP
7A	9 mL	0.001 g	-	0.0075 g
8A	9 mL	-	0.003 g	0.0075 g
9A	9 mL	0.001 g	0.003 g	0.0075 g
GP	9 mL	-	-	0.0075 g

**Table 2:** The data obtained from thermal analysis of the loaded composites: 7A, 8A, 9A, and GP.

Sample	Mass Loss (%)			Residual Mass (%)	Endo	Exo	Exo
	RT–200 °C	200–540 °C	540–900 °C				
GP	7.89	49.51	4.13	38.13	75.2 °C	339.8 °C	470.4 °C
7A	9.29	50.40	3.95	36.40	68.2 °C	336.3 °C	475.3 °C
8A	9.54	52.28	3.84	34.36	64.5 °C	338.7 °C	475.2 °C
9A	9.43	50.52	3.74	36.29	75.5 °C	329.5 °C	465.6 °C

**Table 3:** Comparative analysis with related scaffolds.

Scaffold or Study	Composition	Structure and Porosity	MG-63 Viability	MSC Viability	Distinctive Characteristics
9A (this study)	Collagen–HAp + DOX + CA	20–250-µm pores, interconnected structure	~20% (3 days)	~17%	Selective effect: cytotoxic to MG63, partial protection of BMSCs
Rong et al. (2016) [60]	Collagen–HAp + PLGA/DOX microspheres	100–200-µm pores, 3D interconnected structure	~50% (3 days)	Not reported	Controlled DOX release, in vivo osteointegration, injectable structures
Lu et al. (2021) [61]	Poly(lactic-*co*-glycolic acid)–HAp + DOX + Polydopamine nanofibers	Oriented nanostructure, nanoscale pores	Significantly reduced	Enhanced proliferation	Fibrous structure, regenerative applicability, osteoinductive effects
Wang et al. (2024) [62]	Polydopamine-functionalized calcium-deficient HAp 3D-printed scaffold + DOX	3D-printed scaffold, defined porosity	Considerably reduced	Promoted proliferation	Combined chemo–photothermal therapy, accelerated bone regeneration

## Data Availability

The original contributions presented in the study are included in the article, and further inquiries can be directed to the corresponding author.

## References

[1] The American Cancer Society (2024). American Cancer Society Releases Latest Global Cancer Statistics; Cancer Cases Expected to Rise to 35 Million Worldwide by 2050. News Release.

[2] Bray F., Laversanne M., Sung H., Ferlay J., Siegel R.L., Soerjomataram I., Jemal A. (2024). Global cancer statistics 2022: GLOBOCAN estimates of incidence and mortality worldwide for 36 cancers in 185 countries. CA Cancer J. Clin..

[3] Pullan J.E., Lotfollahzadeh S. (2024). Primary Bone Cancer. [Updated 2024 Mar 20]. StatPearls.

[4] Mirabello L., Troisi R.J., Savage S.A. (2009). International osteosarcoma incidence patterns in children and adolescents, middle ages and elderly persons. Int. J. Cancer.

[5] Misaghi A., Goldin A., Awad M., Kulidjian A.A. (2018). Osteosarcoma: A comprehensive review. SICOT-J..

[6] Wang W., Yeung K.W.K. (2017). Bone grafts and biomaterials substitutes for bone defect repair: A review. Bioact. Mater..

[7] Alonzo M., Primo F.A., Kumar S.A., Mudloff J.A., Dominguez E., Fregoso G., Ortiz N., Weiss W.M., Joddar B. (2021). Bone tissue engineering techniques, advances and scaffolds for treatment of bone defects. Curr. Opin. Biomed. Eng..

[8] Sohn H.S., Oh J.K. (2019). Review of bone graft and bone substitutes with an emphasis on fracture surgeries. Biomater. Res..

[9] Barajas-Pedroza M.A., Rodríguez-Rodríguez R., Nochehdehi A.R., Nemavhola F., Thomas S., Maria H.J. (2024). Development of 3D-printed biocompatible materials for bone substitution. Cartilage Tissue and Knee Joint Biomechanics.

[10] Jaisankar S.N., Haridharan N., Murali A., Sergii P., Špírková M., Mandal A.B., Matějka L. (2014). Single-electron transfer living radical copolymerization of SWCNT-g-PMMA via graft from approach. Polymer.

[11] Koons G.L., Diba M., Mikos A.G. (2020). Materials design for bone-tissue engineering. Nat. Rev. Mater..

[12] Yuan J., Ye Z., Zeng Y., Pan Z., Feng Z., Bao Y., Li Y., Liu X., He Y., Feng Q. (2022). Bifunctional scaffolds for tumor therapy and bone regeneration: Synergistic effect and interplay between therapeutic agents and scaffold materials. Mater. Today Bio..

[13] Chen J., Wen J., Fu Y., Li X., Huang J., Guan X., Zhou Y. (2023). A bifunctional bortezomib-loaded porous nano-hydroxyapatite/alginate scaffold for simultaneous tumor inhibition and bone regeneration. J. Nanobiotechnol..

[14] Yan Z., Deng Y., Huang L., Zeng J., Wang D., Tong Z., Fan Q., Tan W., Yan J., Zang X. (2025). Biopolymer-based bone scaffold for controlled Pt (IV) prodrug release and synergistic photothermal-chemotherapy and immunotherapy in osteosarcoma. J. Nanobiotechnol..

[15] Gong C., Wang J., Tang F., Tong D., Wang Z., Zhou Z., Ruan R., Zhang J., Song J., Yang H. (2024). Bionic Bilayer Scaffold for Synchronous Hyperthermia Therapy of Orthotopic Osteosarcoma and Osteochondral Regeneration. ACS Appl. Mater. Interfaces.

[16] Malla S., Niraula N.P., Singh B., Liou K.K., Sohng J.K. (2010). Limitations in doxorubicin production from *Streptomyces peucetius*. Microbiol. Res..

[17] Park H.-J., Yoon S.-Y., Park J.-N., Suh J.-H., Choi H.-S. (2022). Doxorubicin Induces Bone Loss by Increasing Autophagy through a Mitochondrial ROS/TRPML1/TFEB Axis in Osteoclasts. Antioxidants.

[18] Van der Zanden S.Y., Qiao X., Neefjes J. (2021). New insights into the activities and toxicities of the old anticancer drug doxorubicin. FEBS J..

[19] Serini S., Calviello G. (2024). Potential of Natural Phenolic Compounds against Doxorubicin-Induced Chemobrain: Biological and Molecular Mechanisms Involved. Antioxidants.

[20] Norouzi M., Yathindranath V., Thliveris J.A., Kopec B.M., Siahaan T.J., Miller D.W. (2020). Doxorubicin-loaded iron oxide nanoparticles for glioblastoma therapy: A combinational approach for enhanced delivery of nanoparticles. Sci. Rep..

[21] Cen J., Dai X., Zhao H., Li X., Hu X., Wu J., Duan S. (2023). Doxorubicin-Loaded Liposome with the Function of “Killing Two Birds with One Stone” against Glioma. ACS Appl. Mater. Interfaces.

[22] Kovrlija I., Pańczyszyn E., Demir O., Laizane M., Corazzari M., Locs J., Loca D. (2024). Doxorubicin loaded octacalcium phosphate particles as controlled release drug delivery systems: Physico-chemical characterization, in vitro drug release and evaluation of cell death pathway. Int. J. Pharm..

[23] Liu Y., Raina D.B., Sebastian S., Nagesh H., Isaksson H., Engellau J., Lidgren L., Tägil M. (2021). Sustained and controlled delivery of doxorubicin from an in-situ setting biphasic hydroxyapatite carrier for local treatment of a highly proliferative human osteosarcoma. Acta Biomater..

[24] Rajendran P., Abdelsalam S.A., Renu K., Veeraraghavan V., Ben Ammar R., Ahmed E.A. (2022). Polyphenols as Potent Epigenetics Agents for Cancer. Int. J. Mol. Sci..

[25] Vladu A.F., Ficai D., Ene A.G., Ficai A. (2022). Combination Therapy Using Polyphenols: An Efficient Way to Improve Antitumoral Activity and Reduce Resistance. Int. J. Mol. Sci..

[26] Magnani F., Mattevi A. (2019). Structure and mechanisms of ROS generation by NADPH oxidases. Curr. Opin. Struct. Biol..

[27] Alam M., Ashraf G.M., Sheikh K., Khan A., Ali S., Ansari M.M., Adnan M., Pasupuleti V.R., Hassan M.I. (2022). Potential therapeutic implications of caffeic acid in cancer signaling: Past, present and future. Front. Pharmacol..

[28] Kowalska-Baron A. (2024). Theoretical Insight into Antioxidant Mechanism of Caffeic Acid Against Hydroperoxyl Radicals in Aqueous Medium at Different pH-Thermodynamic and Kinetic Aspects. Int. J. Mol. Sci..

[29] Cortez N., Villegas C., Burgos V., Cabrera-Pardo J.R., Ortiz L., González-Chavarría I., Nchiozem-Ngnitedem V.-A., Paz C. (2024). Adjuvant Properties of Caffeic Acid in Cancer Treatment. Int. J. Mol. Sci..

[30] Lim S.C., Lee T.B., Han S.I. (2024). Caffeic Acid Enhances Anticancer Drug-induced Apoptosis in Acid-adapted HCT116 Colon Cancer Cells. Anticancer Res..

[31] Pelinson L.P., Assmann C.E., Palma T.V., da Cruz I.B.M., Pillat M.M., Mânica A., Stefanello N., Weis G.C.C., Alves A.d.O., de Andrade C.M. (2019). Antiproliferative and apoptotic effects of caffeic acid on SK-Mel-28 human melanoma cancer cells. Mol. Biol. Rep..

[32] Rezaei-Seresht H., Cheshomi H., Falanji F., Movahedi-Motlagh F., Hashemian M., Mireskandari E. (2019). Cytotoxic activity of caffeic acid and gallic acid against MCF-7 human breast cancer cells: An in silico and in vitro study. Avicenna J. Phytomed..

[33] Su Y., Liu Y., Hu X., Lu Y., Zhang J., Jin W., Liu W., Shu Y., Cheng Y.Y., Li W. (2024). Caffeic acid-grafted chitosan/sodium alginate/nanoclay-based multifunctional 3D-printed hybrid scaffolds for local drug release therapy after breast cancer surgery. Carbohydr. Polym..

[34] Colpan R.D., Erdemir A. (2021). Co-delivery of quercetin and caffeic-acid phenethyl ester by polymeric nanoparticles for improved antitumor efficacy in colon cancer cells. J. Microencapsul..

[35] Pagnan A.L., Pessoa A.S., Tokuhara C.K., Fakhoury V.S., Oliveira G.S.N., Sanches M.L.R., Inacio K.K., Ximenes V.F., Oliveira R.C. (2022). Anti-tumour potential and selectivity of caffeic acid phenethyl ester in osteosarcoma cells. Tissue Cell.

[36] Zhu K., Wang W. (2016). Green tea polyphenol EGCG suppresses osteosarcoma cell growth through upregulating miR-1. Tumour Biol. J. Int. Soc. Oncodev. Biol. Med..

[37] Yu Y.-M., Wang L.-Y., Bu F.-Z., Li Y.-T., Wang C., Wu Z.-Y. (2020). The supramolecular self-assembly of 5-fluorouracil and caffeic acid through cocrystallization strategy opens up a new way for the development of synergistic antitumor pharmaceutical cocrystal. CrystEngComm.

[38] Su P., Yang Y., Wang G., Chen X., Ju Y. (2018). CUR Attenuates Resistance to Irinotecan via Induction of Apoptosis of Cancer Stem Cells in Chemoresistant Colon Cancer Cells. Internat. J. Oncol..

[39] Jakobušić Brala C., Karković Marković A., Kugić A., Torić J., Barbarić M. (2023). Combination Chemotherapy with Selected Polyphenols in Preclinical and Clinical Studies—An Update Overview. Molecules.

[40] Cao J., Han J., Xiao H., Qiao J., Han M. (2016). Effect of Tea Polyphenol Compounds on Anticancer Drugs in Terms of Anti-Tumor Activity, Toxicology, and Pharmacokinetics. Nutrients.

[41] Chisholm K., Bray B.J., Rosengren R.J. (2004). Tamoxifen and epigallocatechin gallate are synergistically cytotoxic to MDA-MB-231 human breast cancer cells. Anti-Cancer Drugs.

[42] Tutuianu R., Rosca A.-M., Albu Kaya M.G., Pruna V., Neagu T.P., Lascar I., Simionescu M., Titorencu I. (2020). Mesenchymal stromal cell-derived factors promote the colonization of collagen 3D scaffolds with human skin cells. J. Cell. Mol. Med..

[43] Vladu A.F., Albu Kaya M.G., Truşcă R.D., Motelica L., Surdu V.-A., Oprea O.C., Constantinescu R.R., Cazan B., Ficai D., Andronescu E. (2025). The Role of Crosslinking Agents in the Development of Collagen–Hydroxyapatite Composite Materials for Bone Tissue Engineering. Materials.

[44] Albu M.G. (2011). Collagen Gels and Matrices for Biomedical Applications: The Obtaining and Characterization of Collagen-Based Biomaterials as Support for Local Release.

[45] Albu Kaya M.G., Ferdes M., Kaya D.A., Ghica M.V., Titorencu I., Popa L., Albu L. (2012). Collagen wound dressings with anti-inflamatory activity. Mol. Cryst. Liq. Cryst..

[46] Fecteau K.A., Eiler H. (1998). Evaluation of inhibitory effects of doxorubicin on collagenase using a bovine placentome model. In Vivo.

[47] Mao H., Kawazoe N., Chen G. (2014). Cellular Uptake of Single-Walled Carbon Nanotubes in 3D Extracellular Matrix-Mimetic Composite Collagen Hydrogels. J. Nanosci. Nanotechnol..

[48] Motelica L., Ficai D., Ficai A., Trusca R.D., Ilie C.I., Oprea O.C., Andronescu E. (2020). Innovative antimicrobial chitosan/ZnO/Ag NPs/citronella essential oil nanocomposite—potential coating for grapes. Foods.

[49] Busuioc C., Isopencu G., Banciu A., Banciu D.D., Oprea O., Mocanu A., Deleanu I., Zaulet M., Popescu L., Tanasuica R. (2022). Bacterial cellulose hybrid composites with calcium phosphate for bone tissue regeneration. Int. J. Mol. Sci..

[50] Riaz T., Rabia Z., Faiza Z., Kanwal I., Nawshad M., Zaman S.S., Rahim A., Rizvi S.A.A., Rehman I.U. (2018). FTIR analysis of natural and synthetic collagen. Appl. Spectrosc. Rev..

[51] Tihăuan B.M., Grădișteanu Pîrcălăbioru G., Axinie M., Marinas I.C., Nicoară A.C., Măruțescu L., Oprea O., Matei E., Maier S.S. (2022). Crosslinked Collagenic Scaffold Behavior Evaluation by Physico-chemical, Mechanical and Biological Assessments in an In Vitro Microenvironment. Polymers.

[52] Motelica L., Ficai D., Petrisor G., Oprea O.C., Trușcǎ R.D., Ficai A., Andronescu E., Hudita A., Holban A.M. (2024). Antimicrobial Hydroxyethyl-Cellulose-Based Composite Films with Zinc Oxide and Mesoporous Silica Loaded with Cinnamon Essential Oil. Pharmaceutics.

[53] Sudarsanan K.T., Young R.A. (1969). Significant precision in crystal structural details. Holly Springs hydroxyapatite. Struct. Sci..

[54] Okuda Y., Shigemasa R., Hirota K., Mizutani T. (2022). In Situ Crystallization of Hydroxyapatite on Carboxymethyl Cellulose as a Biomimetic Approach to Biomass-Derived Composite Materials. ACS Omega.

[55] Bai X., Li S., Liu X., An H., Kang X., Guo S. (2022). Caffeic Acid, an Active Ingredient in Coffee, Combines with DOX for Multitarget Combination Therapy of Lung Cancer. J. Agric. Food Chem..

[56] Sandra F., Rizal M.I., Aliwarga C.C., Hadimartana J.C., Celinna M. (2022). Caffeic Acid Induces Apoptosis in MG-63 Osteosarcoma Cells via Protein Kinase C Delta (PKCδ) Translocation and Mitochondrial Membrane Potential Reduction. Indones. Biomed. J..

[57] Valle-Prieto A., Conget P.A. (2010). Human mesenchymal stem cells efficiently manage oxidative stress. Stem Cells Dev..

[58] Yagi H., Tan J., Tuan R.S. (2013). Polyphenols suppress hydrogen peroxide-induced oxidative stress in human bone-marrow derived mesenchymal stem cells. J. Cell Biochem..

[59] Shifa Ul Haq H.M., Ashfaq R., Mehmood A., Shahid W., Ghufran Azam H., Azam M., Tasneem S., Akram S.J., Malik K., Riazuddin S. (2023). Priming with caffeic acid enhances the potential and survival ability of human adipose-derived stem cells to counteract hypoxia. Regen. Ther..

[60] Rong Z.-J., Yang L.-J., Cai B.-T., Zhu L.-X., Cao Y.-L., Wu G.-F., Zhang Z.-J. (2016). Porous nano-hydroxyapatite/collagen scaffold containing drug-loaded ADM–PLGA microspheres for bone cancer treatment. J. Mater. Sci. Mater. Med..

[61] Lu Y., Wan Y., Gan D., Zhang Q., Luo H., Deng X., Li Z., Yang Z. (2021). Enwrapping Polydopamine on Doxorubicin-Loaded Lamellar Hydroxyapatite/Poly(lactic-co-glycolic acid) Composite Fibers for Inhibiting Bone Tumor Recurrence and Enhancing Bone Regeneration. ACS Appl. Bio Mater..

[62] Wang L., Dai Z., Bi J., Chen Y., Wang Z., Sun Z., Ji Z., Wang H., Zhang Y., Wang L. (2024). Polydopamine-functionalized calcium-deficient hydroxyapatite 3D-printed scaffold with sustained doxorubicin release for synergistic chemo-photothermal therapy of osteosarcoma and accelerated bone regeneration. Mater. Today Bio.

